# Mucosal prime with a replicating vaccinia-based vaccine promotes mucosal immunity against SIV

**DOI:** 10.1186/1742-4690-9-S2-P189

**Published:** 2012-09-13

**Authors:** X Tang, Y Du, C Sun, L Chen, L Zhang, Z Chen

**Affiliations:** 1 AIDS Institute, LKS Faculty of Medicine, The University of Hong Kong, Hong Kong, Hong Kong; 2 Institute of Biomedicine and Health, Chinese Academy of Sciences, Guangzhou, China; 3 Comprehensive AIDS Research Center, Tsinghua University, Beijing, China

## Background

We previously demonstrated that vaccine prime with a recombinant replication-competent modified vaccinia Tiantan (rMVTTSIVgpe) was able to enhance the boost effects of a rAd5SIVgpe for eliciting protective immunity against SIV mucosal challenge in rhesus macaques. Whether this heterologous prime and boost regimen is able to elicit potent mucosal immunity specific to SIV remains less understood.

## Methods

Different groups of mice were immunized with the following regiments: rMVTTSIVgpe-rAd5SIVgpe, rAd5SIVgpe-rMVTTSIVgpe and rAd5SIVgpe-rAd5SIVgpe. rMVTTSIVgpe was administrated through intraoral and intranasal routes (ioin) routes whereas rAd5SIVgpe was given through the intramuscular injection (im).

## Results

Consistent with previous findings in macaques, mice immunized with the rMVTTSIVgpe-rAd5SIVgpe regimen generated significantly stronger systemic cellular immune responses as well as serum antibody responses than any other vaccine regimens. Furthermore, as compared with other groups, this rMVTTSIVgpe-rAd5SIVgpe regimen induced significantly higher frequencies of gut-homing CCR9+ Gag-specific CD8+ T cells as well as CCR6+ Gag-specific CD4+ and CD8+ T cells. This regimen also elicited the highest level of CD8+ T cell ELIspot responses against Gag, Pol and Env antigens in mesenteric lymph nodes (mLN). Besides, SIV-specific IgGs could be detected in the rectal wash of mice received rMVTTSIVgpe-rAd5SIVgpe immunization with detectable neutralizing activity.

## Conclusion

These findings demonstrated that mucosal priming with rMVTTSIVgpe significantly promoted mucosal immunity against SIV, which may have implications to the effectiveness of the mucosal rMVTTSIVgpe prime-systemic rAd5SIVgpe boost vacciniation strategy in preventing mucosal infection of SIVmac239 in macaques.

